# Supplementary material for: An open microfluidic coculture model of fibroblasts and eosinophils to investigate mechanisms of airway inflammation
Source: Front Bioeng Biotechnol. 2022 Sep 29;10:993872. doi: 10.3389/fbioe.2022.993872 (PMC9558094; doi:10.3389/fbioe.2022.993872)
Supplement: Supplementary file 1 [file DataSheet1.PDF]

Supplementary Material

1 Supplementary Figures

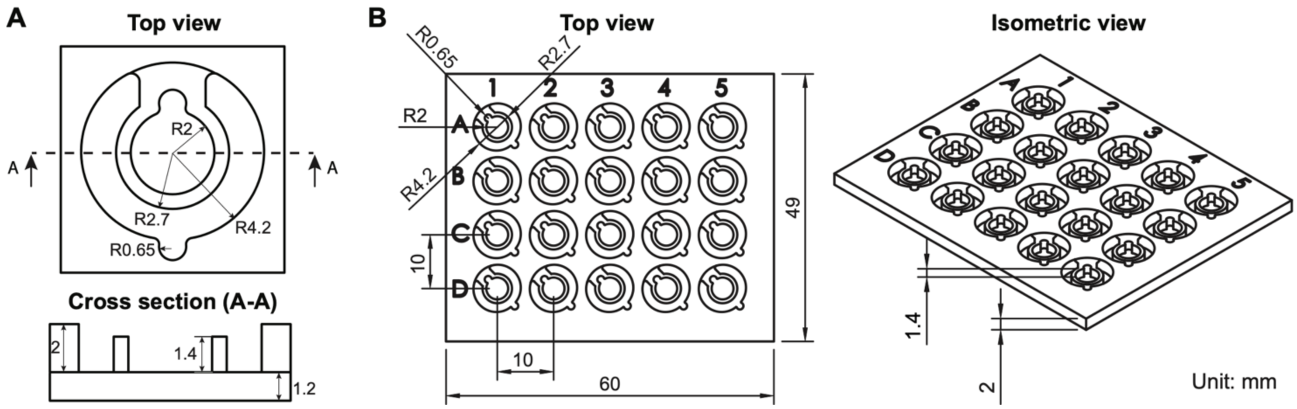

Supplementary Figure 1. Detailed dimensions of the open microfluidic coculture device. **(A)** Dimensions of a single coculture device. Reproduced from Zhang et al., (2020) as allowed per journal policy. Computer aided design file can be found in the Supplementary Material of Zhang et al. **(B)** Dimensions of a milled polystyrene top piece with the coculture devices in a 4×5 array.

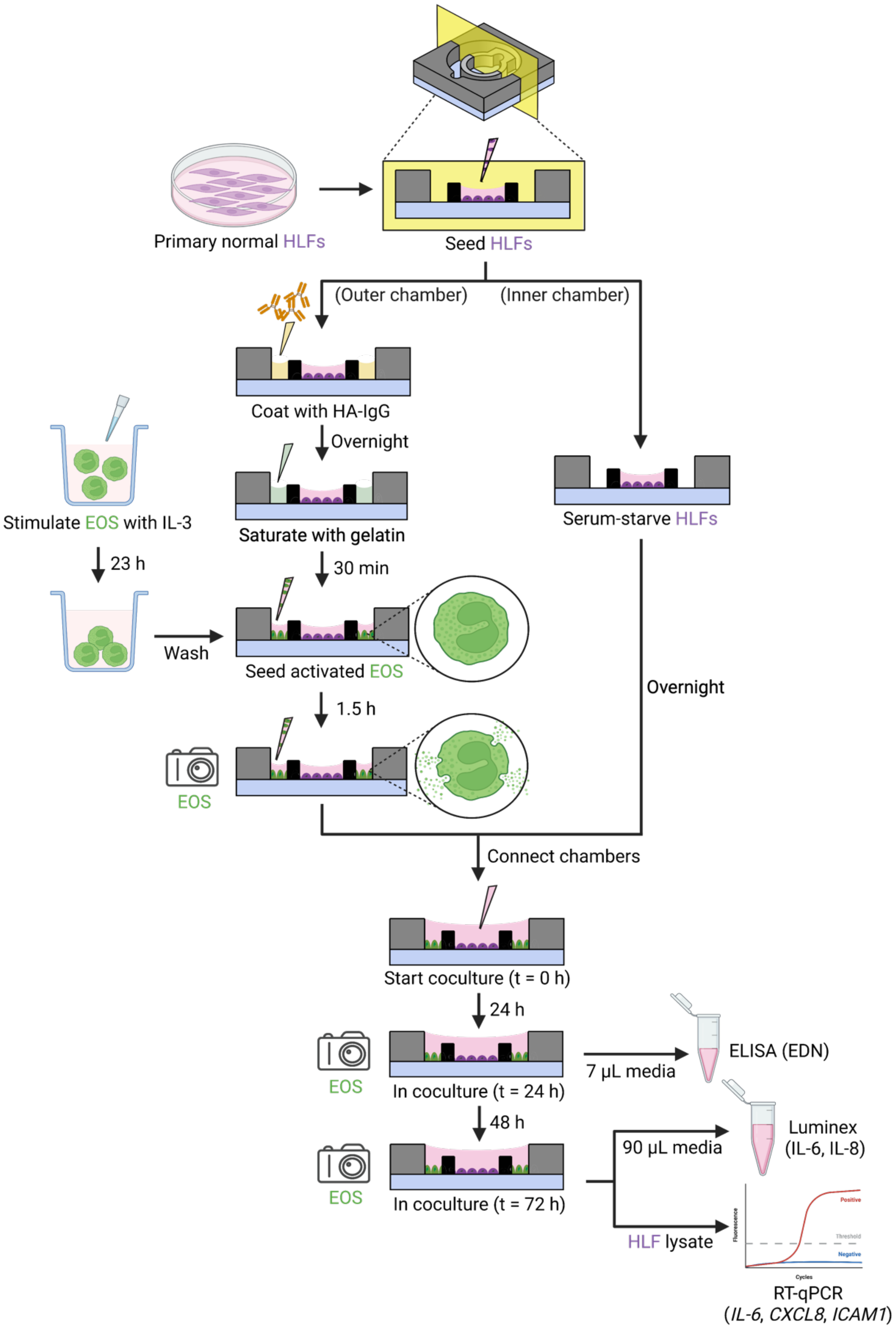

Supplementary Figure 2. Schematic workflow showing cell culture procedures and readouts of the eosinophil (EOS)-human lung fibroblast (HLF) coculture in the open microfluidic coculture device. Created with BioRender.com. HA-IgG: heat-aggregated immunoglobulin G. ELISA: enzyme-linked immunosorbent assay. EDN: eosinophil-derived neurotoxin. IL-6: interleukin-6. IL-8: interleukin-8.

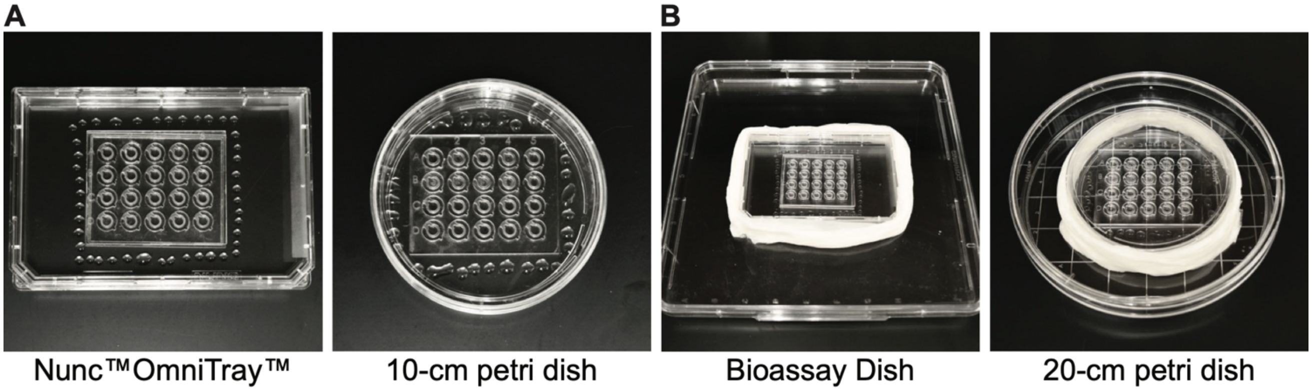

Supplementary Figure 3. Examples of the evaporation control setup for the open microfluidic device. **(A)** Approximately 1 mL of sterile sacrificial water was placed as droplets evenly around the perimeter of the device chip in the primary container (*e.g.,* OmniTray^{TM}, 10-cm petri dish). **(B)** The primary container was placed into a secondary container (*e.g.,* 30×30 cm bioassay dish or 20-cm petri dish). The perimeter of the primary container was wrapped tightly with Kimwipes soaked with sterile water. In this study, we used OmniTray^{TM} and Bioassay Dish as the primary and secondary container, respectively.

In the next pages, we show a complete collection of results from all donor pairs (including the 2 pairs shown in the main manuscript). Figure captions might be on a different page due to large figures.

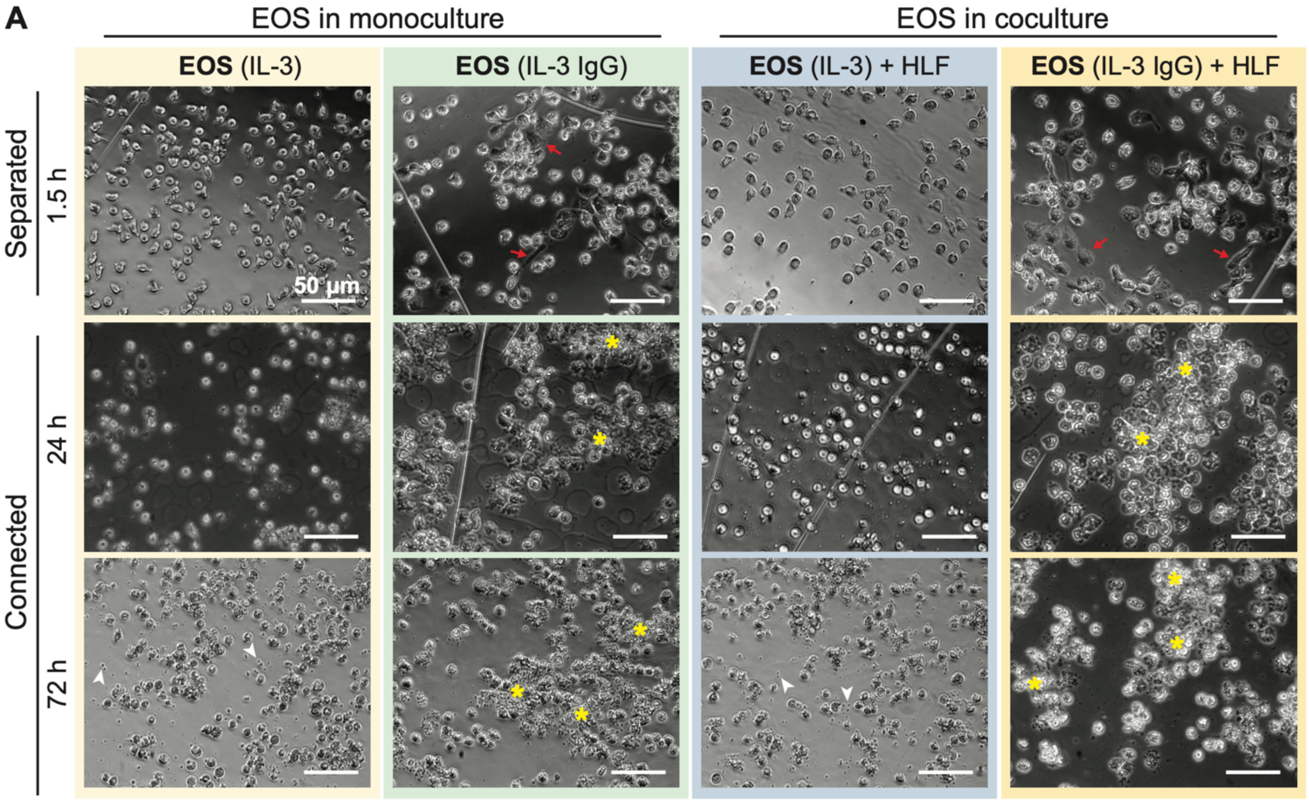

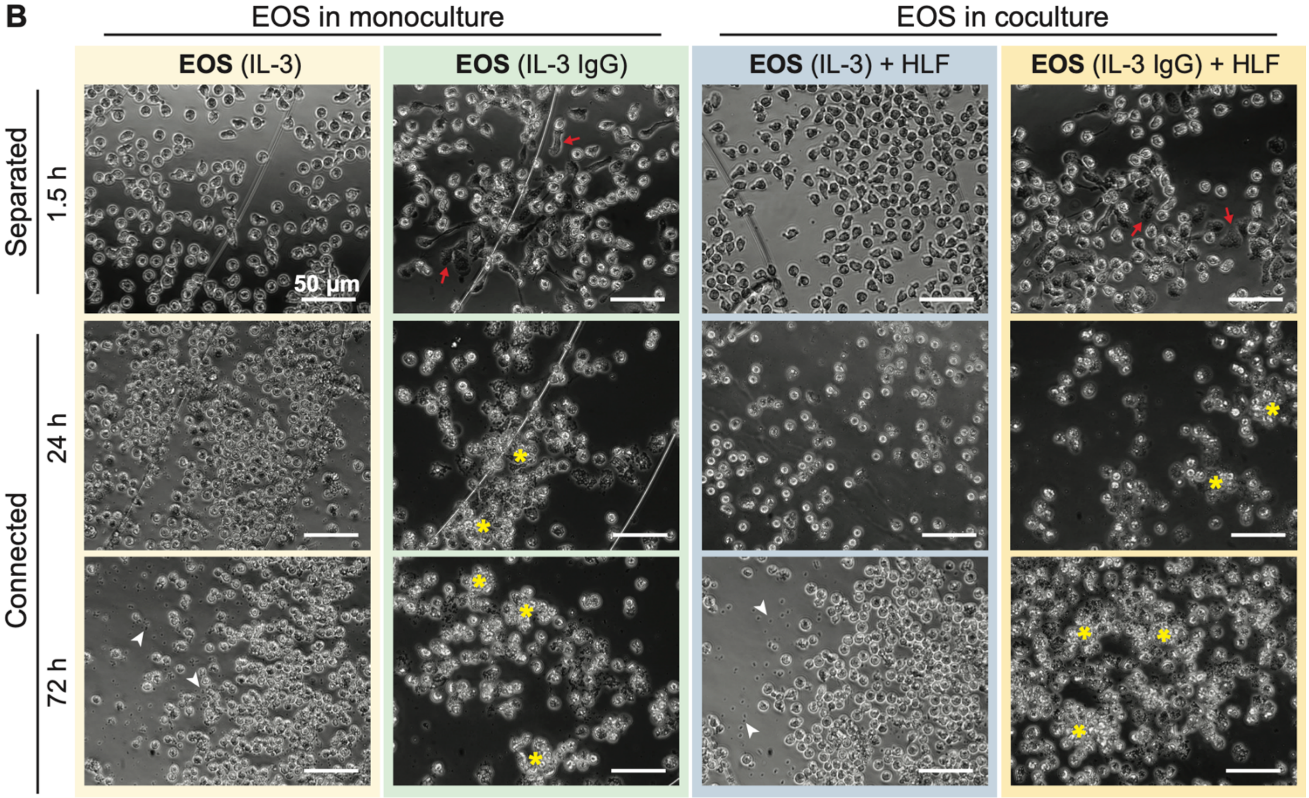

(continued next page)

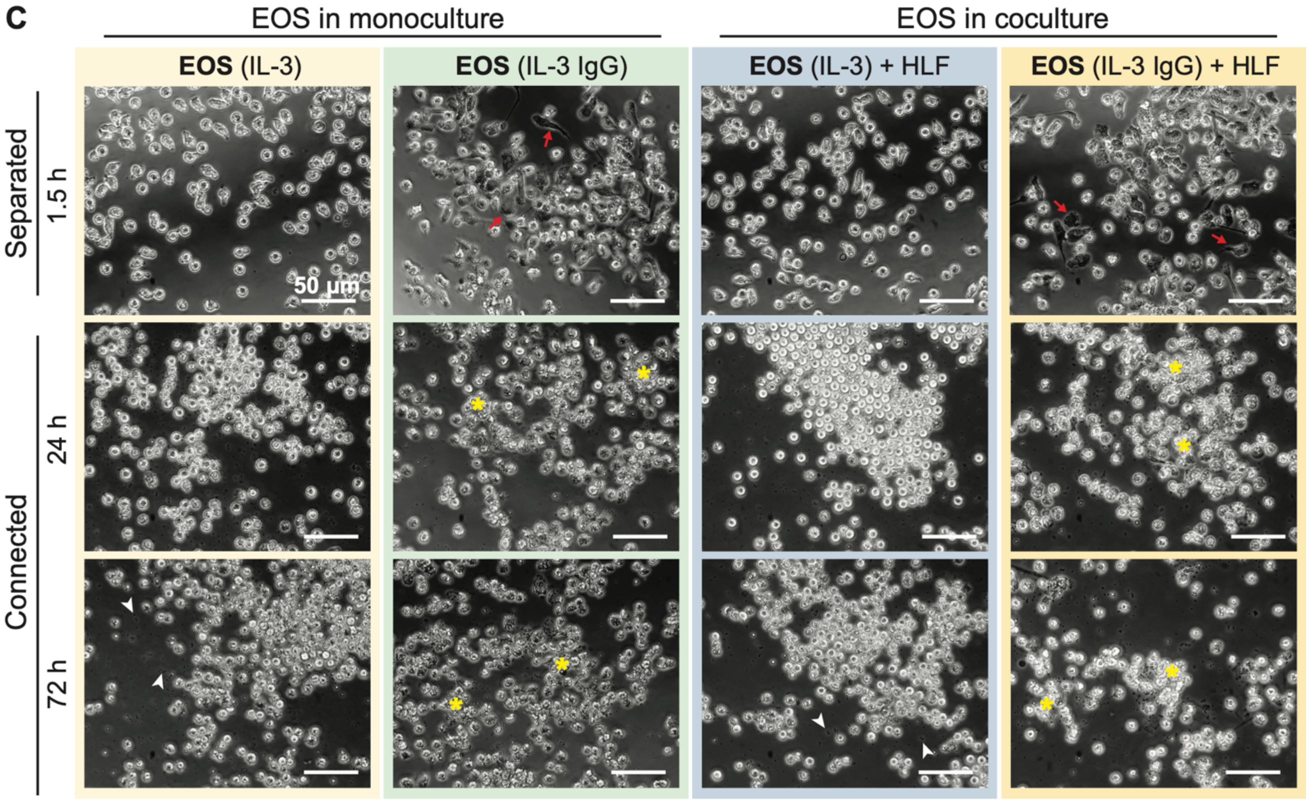

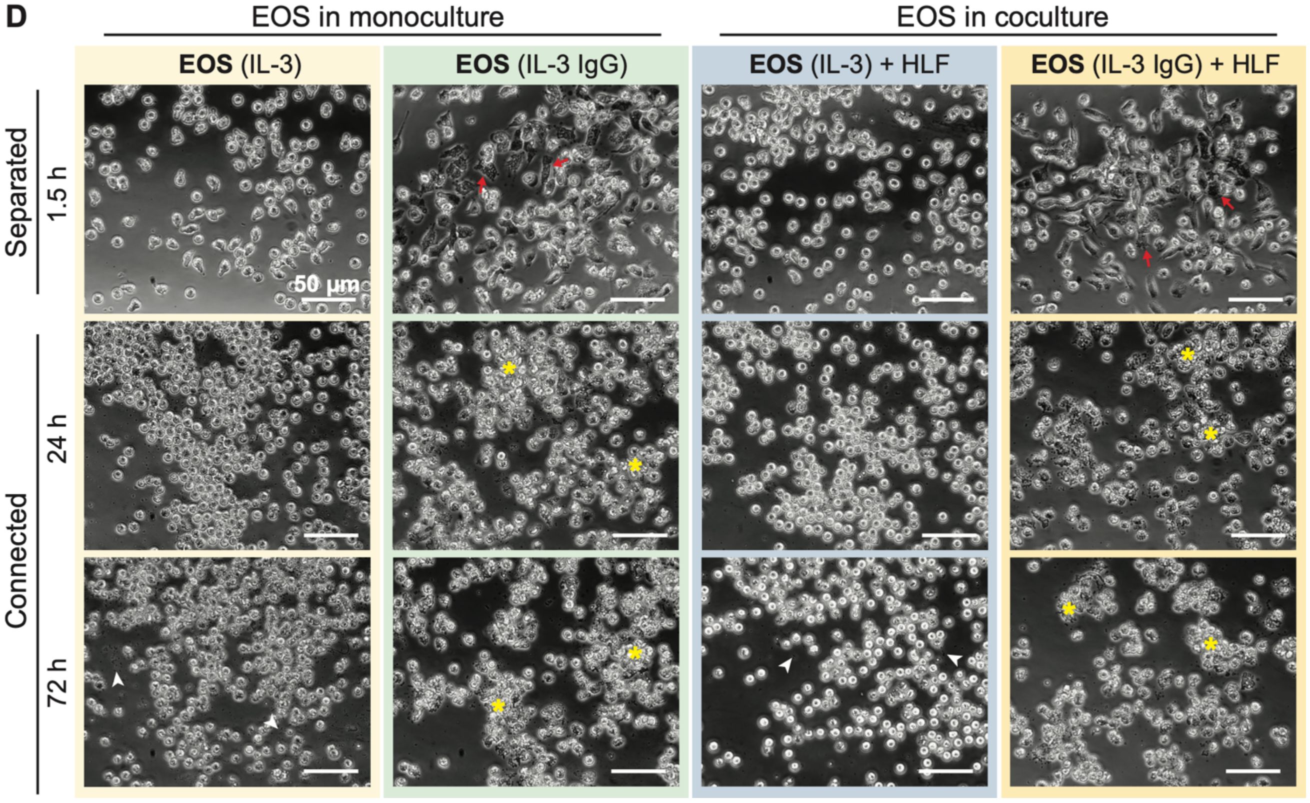

(continued next page)

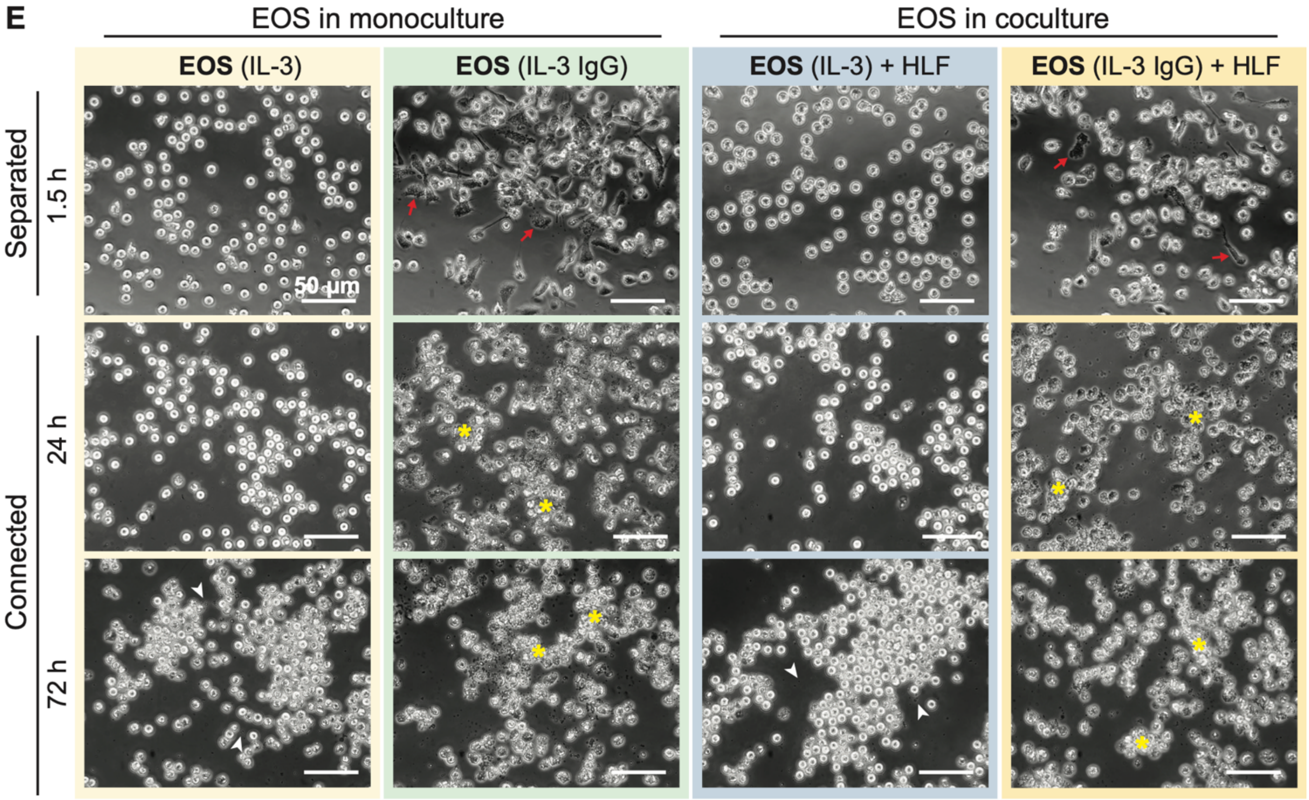

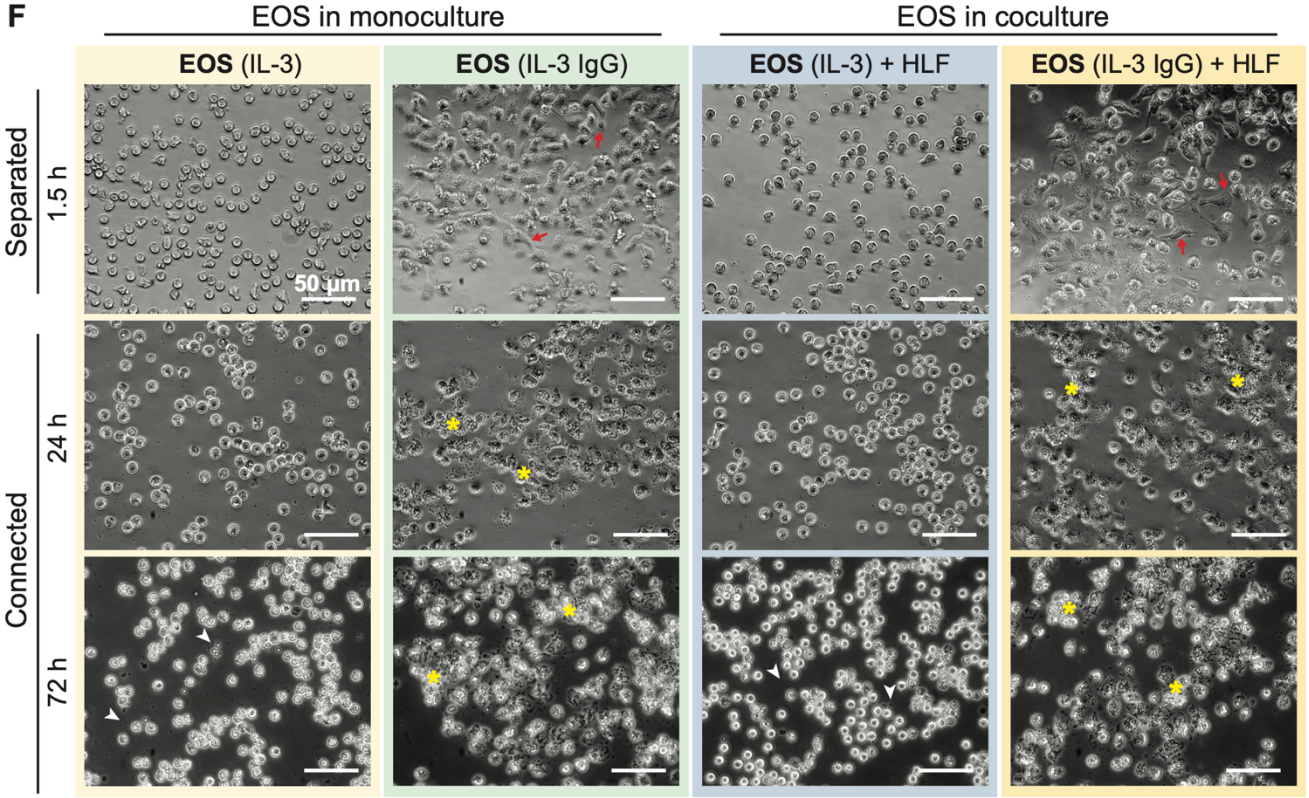

(continued next page)

Supplementary Figure 4. Phase contrast images of eosinophils activated with IL-3 ("IL-3") and in some conditions also seeded onto HA-IgG ("IL-3 IgG") in coculture devices at the indicated times. Representative images from 6 donor pairs are shown: **(A)** eosinophil donor 1 and HLF donor 1, **(B)** eosinophil donor 1 and HLF donor 2, **(C)** eosinophil donor 2 and HLF donor 1, **(D)** eosinophil donor 2 and HLF donor 2, **(E)** eosinophil donor 3 and HLF donor 1, **(F)** eosinophil donor 3 and HLF donor 2. The chambers remained separated for 1.5 h and then were connected for up to 72 h. Red arrows show eosinophils spreading on HA-IgG. Yellow asterisks show aggregates of eosinophil cell debris. White arrowheads show eosinophil cell-free granules. Images are representative of 4 culture replicates.

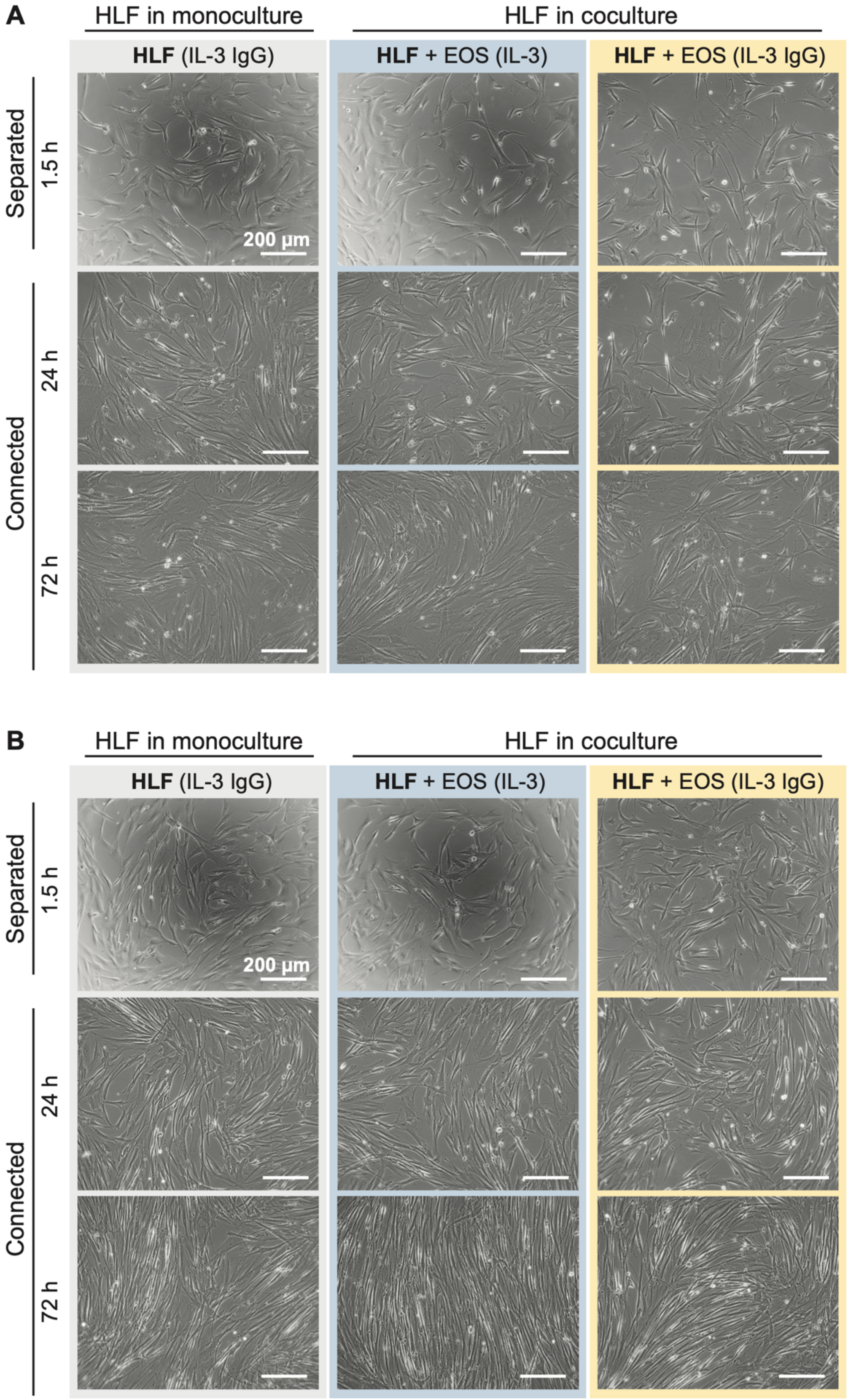

(continued next page)

Supplementary Figure 5. Phase contrast images of HLFs in coculture devices at the indicated times. Representative images from 2 donor pairs are shown: **(A)** eosinophil donor 2 and HLF donor 1, **(B)** eosinophil donor 2 and HLF donor 2. The chambers remained separated for 1.5 h and then were connected for up to 72 h. Images are representative of 4 culture replicates, as well as 3 eosinophil donors and 2 HLF donors.

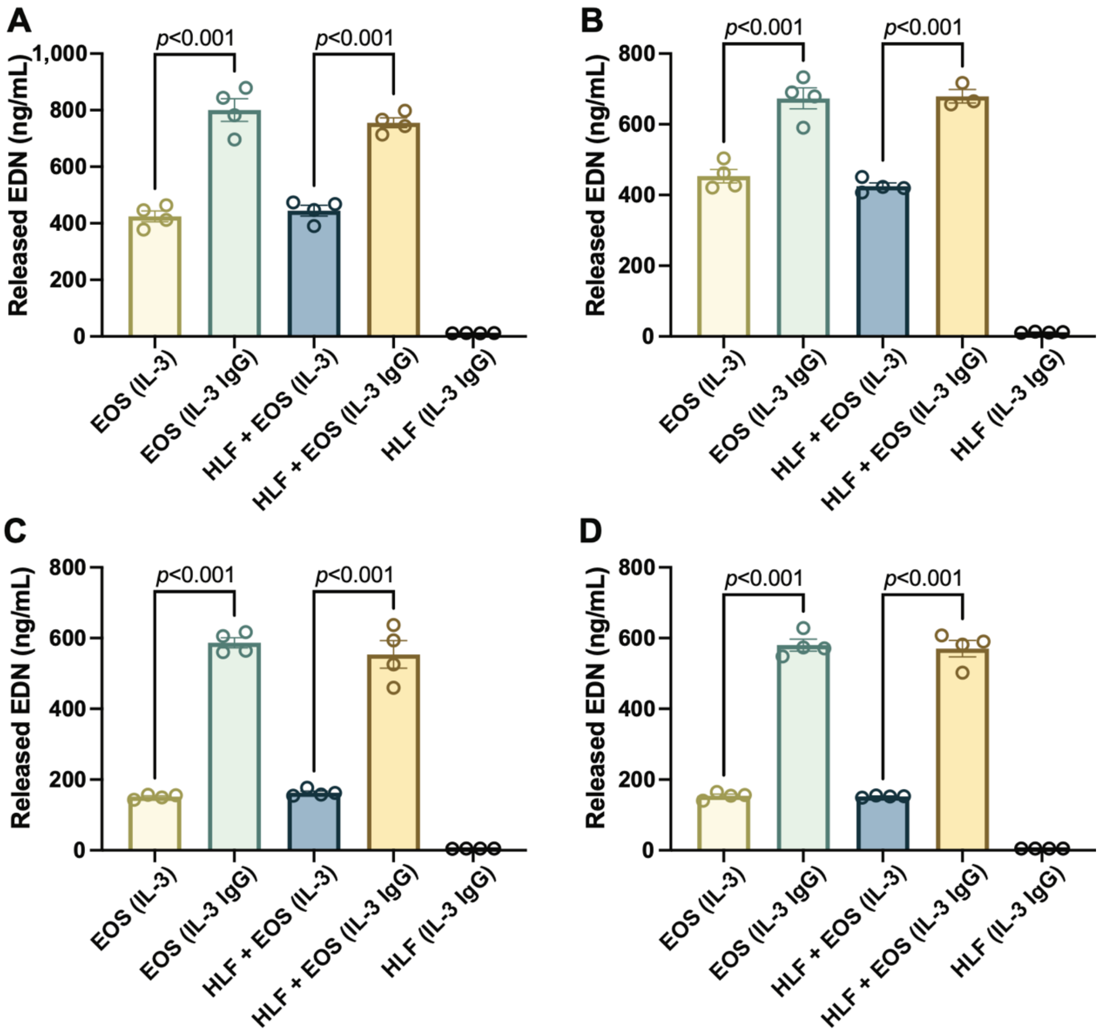

Supplementary Figure 6. Levels of released eosinophil-derived neurotoxin (EDN) in culture media after 24 h measured by ELISA. Eosinophils release the highest level of EDN under IL-3 IgG conditions regardless of coculture or monoculture. Results from 4 donor pairs are shown: **(A)** eosinophil donor 1 and HLF donor 1, **(B)** eosinophil donor 1 and HLF donor 2, **(C)** eosinophil donor 3 and HLF donor 1, **(D)** eosinophil donor 3 and HLF donor 2. The 2 donor pairs with eosinophil donor 2 were excluded due to protocol differences in ELISA. Data are expressed as mean ± SEM (n=4); each point is one culture replicate. Data were analyzed using one-way ANOVA, followed by Holm-Šídák's multiple comparisons test.

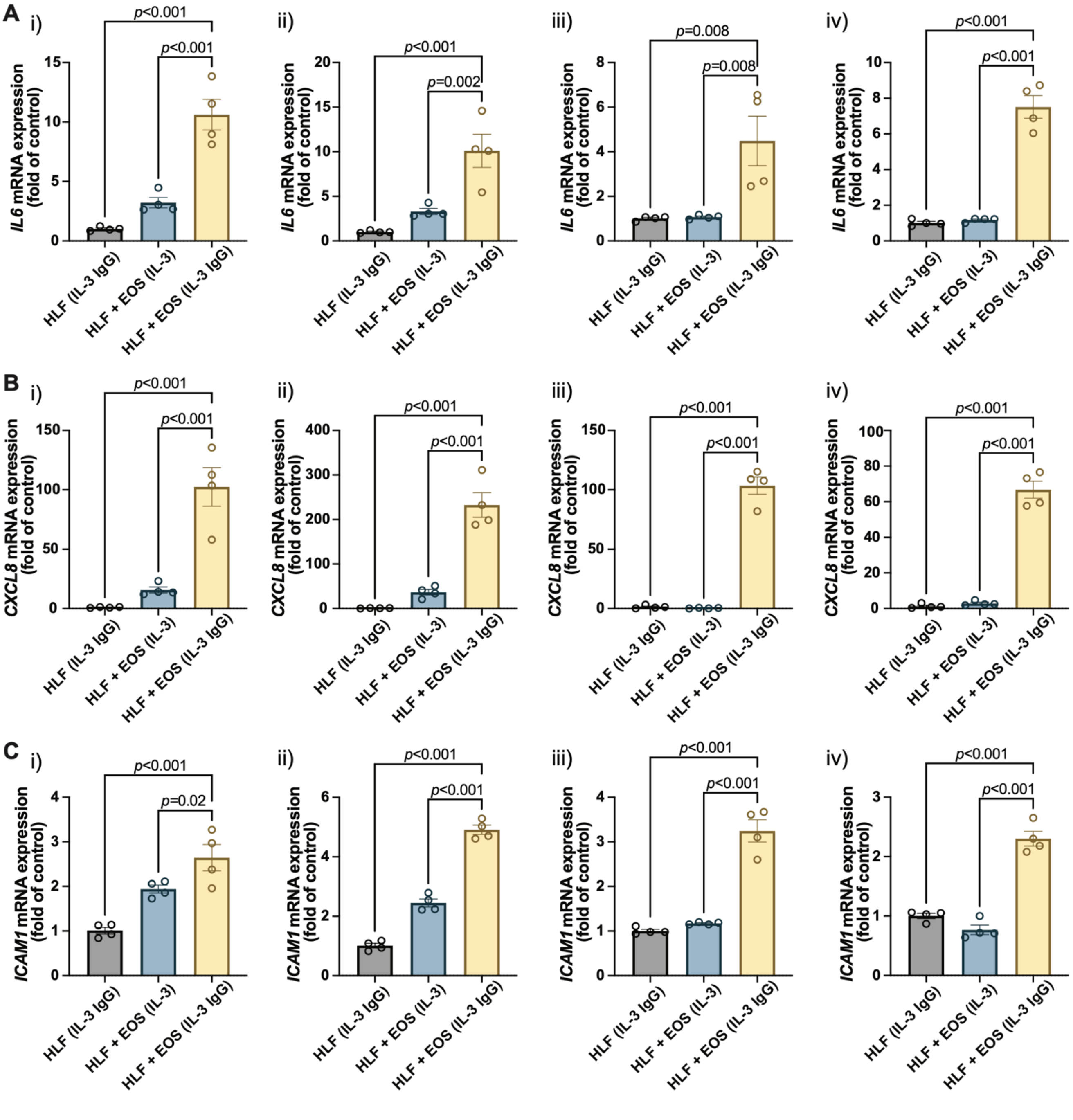

Supplementary Figure 7. Proinflammatory genes are upregulated in HLFs cocultured with degranulating eosinophils. HLFs in coculture with degranulating eosinophils ("HLF + EOS (IL-3 IgG)") for 72 h showed the highest mRNA level upregulation of **(A)** *IL6*, **(B)** *CXCL8*, and **(C)** *ICAM1*. Results from 4 donor pairs are shown: **(i)** eosinophil donor 1 and HLF donor 1, **(ii)** eosinophil donor 1 and HLF donor 2, **(iii)** eosinophil donor 2 and HLF donor 1, **(iv)** eosinophil donor 2 and HLF donor 2. Data from the 2 donor pairs with eosinophil donor 3 were not included because the samples were used to develop a separate readout. Data are expressed as mean ± SEM (n=4); each point is one culture replicate. Data were analyzed using one-way ANOVA, followed by Holm-Šídák's multiple comparisons test.

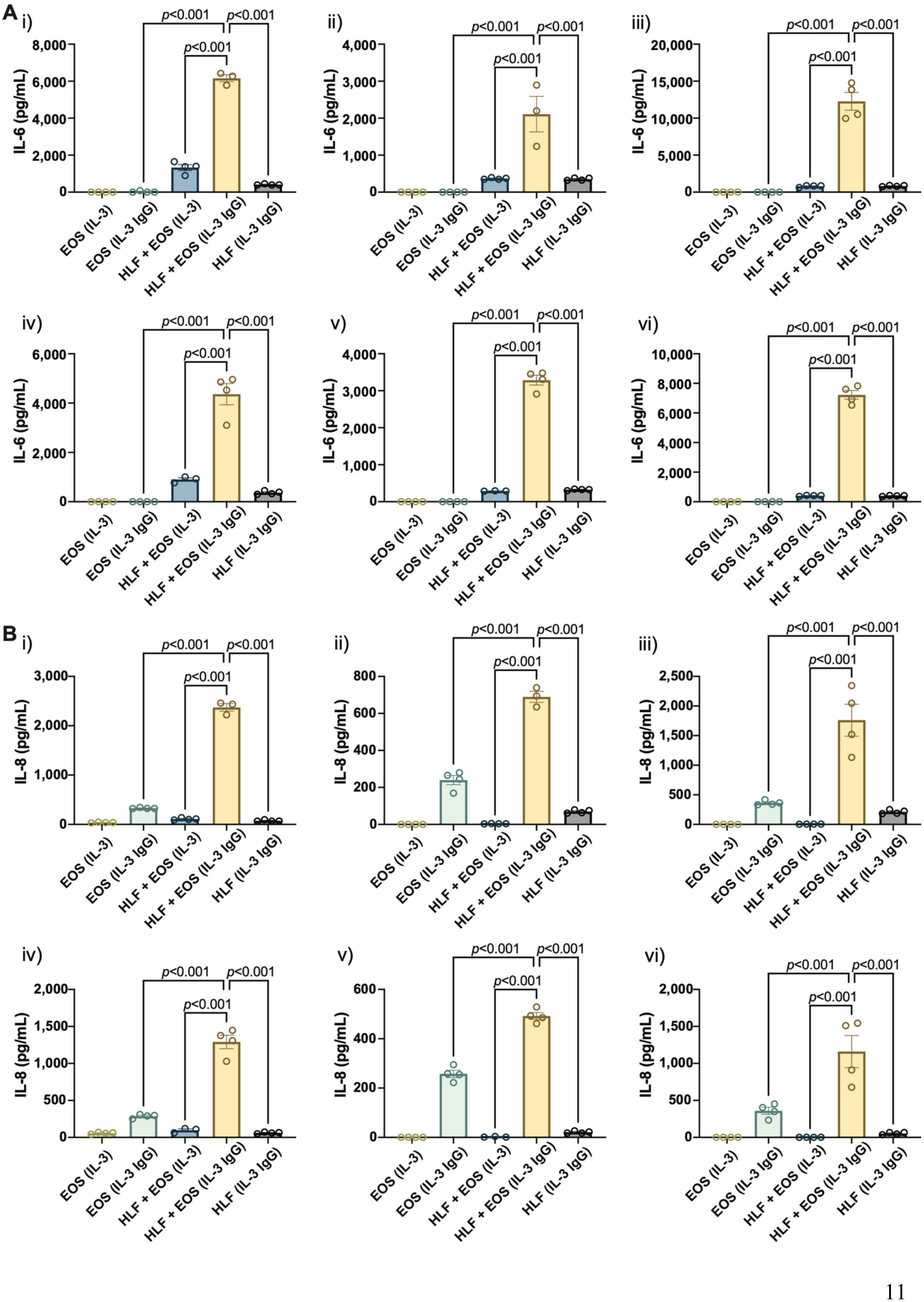
A
i)
ii)
iii)
iv)
v)
vi)
B
i)
ii)
iii)
iv)
v)
vi)
IL-6 (pg/mL)
IL-8 (pg/mL)
p<0.001
EOS (IL-3)
EOS (IL-3 IgG)
HLF + EOS (IL-3)
HLF + EOS (IL-3 IgG)
HLF (IL-3 IgG)

Supplementary Figure 8. The coculture of HLFs with degranulating eosinophils releases the highest levels of proinflammatory cytokines compared to HLF and eosinophil monocultures and other control conditions. Levels of soluble factors, **(A)** IL-6 and **(B)** IL-8, were measured in the conditioned media after 72 h in culture. Results from 6 donor pairs are shown: **(i)** eosinophil donor 1 and HLF donor 1, **(ii)** eosinophil donor 2 and HLF donor 1, **(iii)** eosinophil donor 3 and HLF donor 1, **(iv)** eosinophil donor 1 and HLF donor 2, **(v)** eosinophil donor 2 and HLF donor 2, **(vi)** eosinophil donor 3 and HLF donor 2. Data are expressed as mean ± SEM (n=3-4); each point is one culture replicate. Data were analyzed using one-way ANOVA, followed by Holm-Šídák's multiple comparisons test.

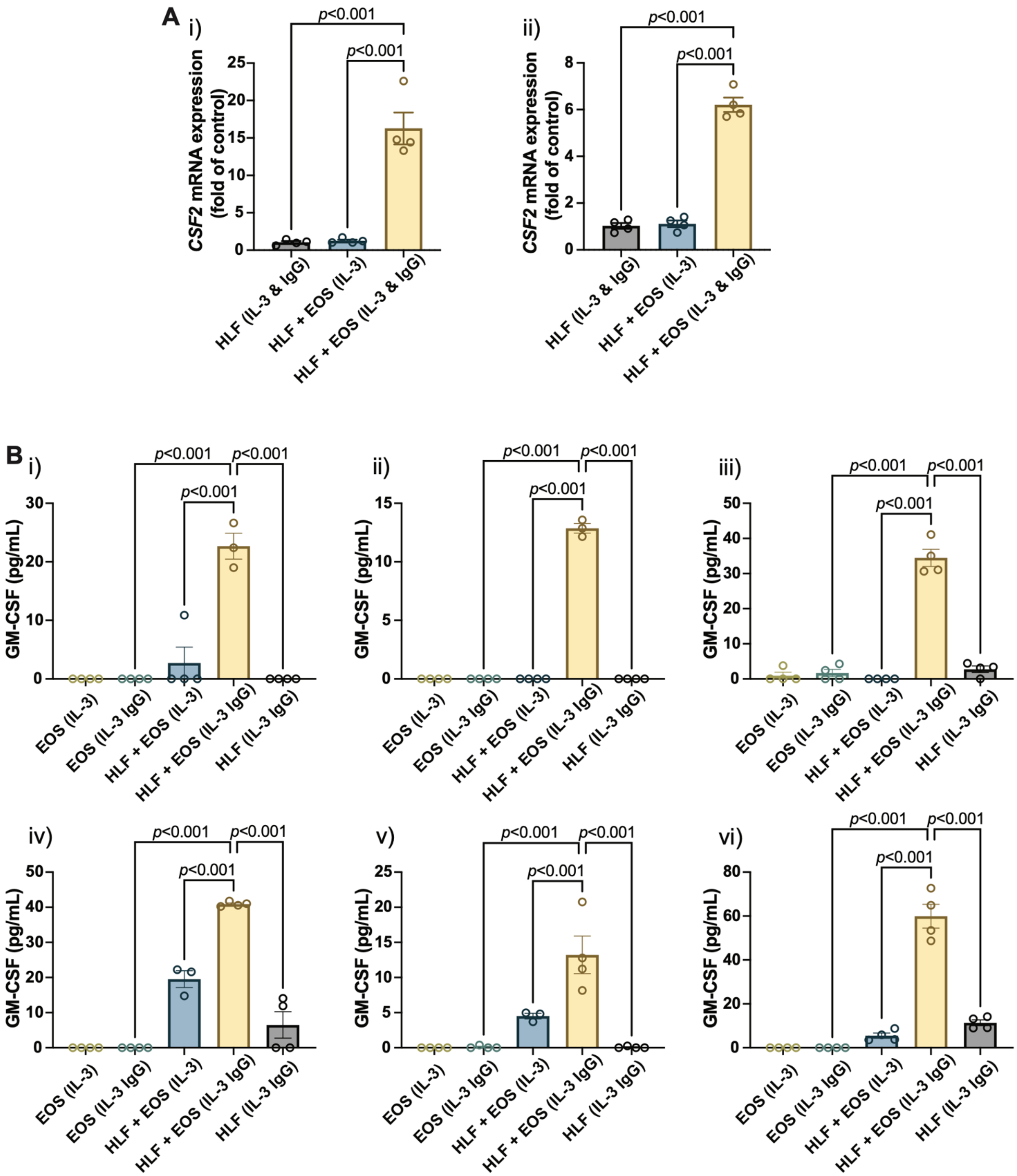

Supplementary Figure 9. The coculture of HLFs with degranulating eosinophils induces the highest levels of expression and secretion of *CSF2*/GM-CSF than other control conditions. **(A)** mRNA levels of *CSF2* in HLFs after 72 h in culture. Results from 2 donor pairs are shown: **(i)** eosinophil donor 2 and HLF donor 1, **(ii)** eosinophil donor 2 and HLF donor 2. Data from the 4 donor pairs with eosinophil donors 1 and 3 were not included, because the samples were limited and used up to develop a separate readout. **(B)** Levels of GM-CSF measured in the conditioned media after 72 h in culture. Results from 6 donor pairs are shown: **(i)** eosinophil donor 1 and HLF donor 1, **(ii)**

eosinophil donor 2 and HLF donor 1, **(iii)** eosinophil donor 3 and HLF donor 1, **(iv)** eosinophil donor 1 and HLF donor 2, **(v)** eosinophil donor 2 and HLF donor 2, **(vi)** eosinophil donor 3 and HLF donor 2. Data are expressed as mean ± SEM (n=3-4); each point is one culture replicate. Data were analyzed using one-way ANOVA, followed by Holm-Šídák's multiple comparisons test.

2 Supplementary Tables

Supplementary Table 1. Calculation of eosinophil seeding densities in 96-well plates (Esnault et al., 2017) or outer chambers of the microfluidic coculture device. The surface area seeding density was kept consistent in both cultureware.

	**96-well plate**	**Device outer chamber**
Media volume	100 µL	36 µL
Seeding area	32 mm^2	29.3 mm^2
Volume density	1×10^6 cells/mL	2.6×10^6 cells/mL
Area density	3125 cells/mm^2	
Total cell number per well	1×10^5 cells	9.2×10^4 cells

Supplementary Table 2. Forward and reserve sequences of primers used in reverse transcription quantitative polymerase chain reaction (RT-qPCR). *GUSB*: glucuronidase β. *IL6*: interleukin 6. *CXCL8*: C-X-C motif chemokine ligand 8. *ICAM1*: intercellular adhesion molecule 1. *CSF2*: colony stimulating factor 2.

**Primer**	**Forward**	**Reverse**
GUSB	CAGGACCTGCGCACAAGAG	AGCGTGTCGACCCCATTC
IL6	TGCAGATGAGTACAAAAGTCCTGAT	GTGGTTATTGCATCTAGATTCTTTGC
CXCL8	CTTGGCAGCCTTCCTGATTT	TTCTTTAGCACTCCTTGGCAAAA
ICAM1	GCCAGGAGACACTGCAGACA	TGGCTTCGTCAGAATCACGTT
CSF2	TGAATGAAACAGTAGAAGTCATCTCAGAAAT	GCTCCAGGCGGGTCTGTA

3 Reference

Esnault, S., Johansson, M. W., Kelly, E. A., Koenderman, L., Mosher, D. F., and Jarjour, N. N. (2017). IL-3 up-regulates and activates human eosinophil CD32 and αMβ2 integrin causing degranulation. *Clin. Exp. Allergy* 47, 488–498. doi:10.1111/cea.12876.

Zhang, T., Lih, D., Nagao, R. J., Xue, J., Berthier, E., Himmelfarb, J., et al. (2020). Open microfluidic coculture reveals paracrine signaling from human kidney epithelial cells promotes kidney specificity of endothelial cells. *Am. J. Physiol. - Ren. Physiol.* 319, F41–F51. doi:10.1152/ajprenal.00069.2020.
